# ΔF508 CFTR Surface Stability Is Regulated by DAB2 and CHIP-Mediated Ubiquitination in Post-Endocytic Compartments

**DOI:** 10.1371/journal.pone.0123131

**Published:** 2015-04-16

**Authors:** Lianwu Fu, Andras Rab, Li ping Tang, Zsuzsa Bebok, Steven M. Rowe, Rafal Bartoszewski, James F. Collawn

**Affiliations:** 1 Department of Cell, Developmental and Integrative Biology, University of Alabama at Birmingham, Birmingham, Alabama, United States of America; 2 Department of Medicine, University of Alabama at Birmingham, Birmingham, Alabama, United States of America; 3 Gregory Fleming James Cystic Fibrosis Research Center, University of Alabama at Birmingham, Birmingham, Alabama, United States of America; 4 Department of Biology and Pharmaceutical Botany, Medical University of Gdansk, Gdansk, Poland; Medical University of Gdańsk, POLAND

## Abstract

The ΔF508 mutant form of the cystic fibrosis transmembrane conductance regulator (ΔF508 CFTR) that is normally degraded by the ER-associated degradative pathway can be rescued to the cell surface through low-temperature (27°C) culture or small molecular corrector treatment. However, it is unstable on the cell surface, and rapidly internalized and targeted to the lysosomal compartment for degradation. To understand the mechanism of this rapid turnover, we examined the role of two adaptor complexes (AP-2 and Dab2) and three E3 ubiquitin ligases (c-Cbl, CHIP, and Nedd4-2) on low-temperature rescued ΔF508 CFTR endocytosis and degradation in human airway epithelial cells. Our results demonstrate that siRNA depletion of either AP-2 or Dab2 inhibits ΔF508 CFTR endocytosis by 69% and 83%, respectively. AP-2 or Dab2 depletion also increases the rescued protein half-life of ΔF508 CFTR by ~18% and ~91%, respectively. In contrast, the depletion of each of the E3 ligases had no effect on ΔF508 CFTR endocytosis, whereas CHIP depletion significantly increased the surface half-life of ΔF508 CFTR. To determine where and when the ubiquitination occurs during ΔF508 CFTR turnover, we monitored the ubiquitination of rescued ΔF508 CFTR during the time course of CFTR endocytosis. Our results indicate that ubiquitination of the surface pool of ΔF508 CFTR begins to increase 15 min after internalization, suggesting that CFTR is ubiquitinated in a post-endocytic compartment. This post-endocytic ubiquination of ΔF508 CFTR could be blocked by either inhibiting endocytosis, by siRNA knockdown of CHIP, or by treating cells with the CFTR corrector, VX-809. Our results indicate that the post-endocytic ubiquitination of CFTR by CHIP is a critical step in the peripheral quality control of cell surface ΔF508 CFTR.

## Introduction

The cystic fibrosis transmembrane conductance regulator (CFTR) is a cAMP-activated chloride and bicarbonate channel that is important for ion balance and fluid transport in a number of epithelial cell types (reviewed in [1]). CFTR is expressed at the apical surface of human airway epithelia and loss of CFTR function in cystic fibrosis (CF) results in mucus accumulation, reoccurring bacterial infections, respiratory inflammation, and declining lung function [2, 3]. Although more than 2000 mutations have been described for the *CFTR* gene, one mutation, ΔF508 CFTR, is found in more than 90% of the patients and therefore has become a primary target for testing therapeutic interventions [4, 5].

ΔF508 CFTR fails to fold properly during biosynthesis in the ER and is retrotranslocated and rapidly degraded by the ER-associated degradative pathway [6]. The mutation appears to be temperature-sensitive since culturing cells expressing ΔF508 CFTR at 26–30°C for 24 to 48 hours results in delivery of some ΔF508 CFTR to the cell surface [7]. However, this cell surface ΔF508 CFTR is unstable at 37°C and is rapidly internalized and degraded in the lysosomal compartment [8–12]. Examining the quality control machinery in the ER has revealed that a number of chaperones, co-chaperones, and E3 ubiquitin-ligases (CHIP and Rma1) are important for ΔF508 CFTR degradation [13–16]. Analysis of the peripheral quality control machinery at the cell surface in HeLa cells revealed that siRNA knockdown of the E3 ligase CHIP increases rescued ΔF508 CFTR surface stability [11], suggesting that low-temperature rescued ΔF508 CFTR is misfolded at 37°C.

To internalize cell surface proteins, adaptor complexes bind to clathrin and simultaneously bind to the cytoplasmic tails of the cell surface molecules to promote protein clearance from the cell surface. Interestingly, c-Cbl, an E3 ligase, has been implicated as one of three adaptors (c-Cbl, Dab2, and AP-2) that promote wild type CFTR internalization through clathrin-coated pits [17–23]. Since ubiquitination acts as a signal for the internalization and sorting of plasma membrane proteins, particularly receptor tyrosine kinases such as the epidermal growth factor receptor [24, 25], it is conceivable that E3 ligases such as c-Cbl, also mediate CFTR internalization and lysosomal degradation. Indeed, one study in airway epithelial cells suggested that c-Cbl mediated both endocytosis and lysosomal targeting of wild type CFTR in airway epithelial cells, although its effect on CFTR endocytosis was reported to be independent of its E3 ligase activity [17]. Our own investigation indicated that c-Cbl had no effect on wild type CFTR endocytosis but did increase CFTR stability [23]. To complicate matters further, it has been proposed that the specific adaptors controlling CFTR endocytosis are tissue-specific [26].

In the present studies, we examined steps that are involved in the rapid turnover of rescued ΔF508 CFTR (rΔF508 CFTR) from the cell surface. We tested the role of two endocytosis adaptor complexes and three E3 ligases on the endocytosis and stability of rΔF508 CFTR. We found that the two adaptors, AP-2 and Dab2, were necessary for rΔF508 CFTR internalization but none of the E-3 ligases, c-Cbl, CHIP and Nedd4-2, had any effect at this initial step in airway epithelial cells. We also show that ubiquitination of rΔF508 CFTR occurs after endocytosis and is mediated by CHIP, and Dab2 plays a role in targeting the ubiquitinated rΔF508 CFTR to the lysosome. We also show that the investigational CFTR corrector Lumacaftor (VX-809) inhibits CFTR ubiquitination and increases rΔF508 CFTR cell surface stability. Our results suggest that Dab2 and CHIP act in concert to target misfolded rΔF508 CFTR to the lysosome.

## Materials and Methods

### Cell culture

CFBE41o-ΔF (CFBE41o- cells expressing ΔF508 CFTR) cells were routinely maintained in DMEM (Dulbecco’s modified Eagle’s medium) and Ham’s F12 medium (50:50, v/v; Life Technologies) at 37°C in a humidified incubator containing 5% CO_2_ as described previously [27]. For low-temperature and delivery of ΔF508-CFTR to the plasma membrane, cells were incubated at 27°C for 24 h or 48 h prior to experiments as indicated. For polarized monolayers, CFBE41o-ΔF cells were seeded onto membrane inserts (Costar, Corning) and cultured on an air-liquid interface for 4 to 5 days before analysis as described previously [10].

### Antibodies and chemicals

Anti-CFTR, mouse monoclonal antibodies (clone 24–1 (ATCC) and MM13-4 (Millipore)) were used as described previously [23]. Mouse anti-AP50/μ2 monoclonal antibody was purchased from BD Transduction Laboratories and used in 1:500 dilutions for immunoblotting. Rabbit polyclonal antibody to mannose-6-phosphate receptor, c-Cbl and Nedd4-2 were purchased from Abcam. Rabbit polyclonal antibody against Dab2 was from Santa Cruz Biotechnology and used in 1:200 dilution. Rabbit polyclonal, anti-CHIP antibody was from Thermo Scientific. Polyclonal anti-β-actin antibody was purchased from Sigma-Aldrich. Alexa Fluor 488-labeled goat anti-mouse IgG antibody and Alexa Fluor 594-labeled goat anti-rabbit IgG antibody were from Invitrogen and used in 1:200 dilution. HRP-conjugated goat anti-mouse IgG antibody and anti-rabbit IgG antibody were from Bio-Rad Laboratories. The SuperSignal West Pico chemiluminescence substrate was purchased from Pierce Chemical Co. The ΔF508 CFTR corrector VX-809 was purchased from Selleck Chemicals. All other chemicals were from Sigma-Aldrich or Thermo Scientific.

### siRNA depletion of Dab2, μ2, c-Cbl, CHIP and Nedd4-2

siRNA duplexes corresponding to non-conserved regions of human Dab2, μ2 and c-Cbl were purchased from Qiagen Inc. siRNA oligos for Nedd4-2 knock-down were purchased from Ambion^®^ (Life technologies). Human STUB1 (CHIP) was depleted by using siRNA oligos from Dharmacon as described previously [11]. The specific sequence 5’-TAGAGCATGAACATCCAGTAA-3’ was chosen as a target for the small interfering RNA (siRNA) depletion of Dab2. The targeting sequence for μ2 knockdown was 5’-TGCCATCGTGTGGAAGATCAA-3’. The targeting sequence for c-Cbl was 5’-CCCGCCGAACUCUCAGAUATT-3’. The targeting sequence for Nedd4-2 knockdown was 5’-CCACAACACAAAGTCACACAG-3’. The double-stranded non-silencing control siRNA sequence 5’-AATTCTCCGAACGTGTCACGT-3’, which has no significant homology to any other genes, was also purchased from Qiagen Inc. Transfection of siRNA oligos was performed using siLentFect lipid reagent (Bio-Rad Laboratories) according to the manufacturer’s instructions. Briefly, cells at ~70–80% confluence were transfected with the optimized transfection mixture. After 24 h incubation at 37°C, the transfection mixture was replaced with fresh cell culture medium. Experiments were conducted 3 to 6 days after transfection. The depletion efficiency of individual genes was assessed by Western blotting in each experiment.

### Western blotting

CFTR, μ2, Dab2, c-Cbl, CHIP and Nedd4-2 protein levels in control or siRNA-depleted samples were determined as described previously [23]. In brief, about 25 μg cell lysates from each sample were resolved by SDS-PAGE and transferred to PDVF membrane followed by blotting with specific primary antibody and HRP-labeled secondary antibody. The membrane was then developed using chemiluminescent substrate (SuperSignal West Pico, Pierce) and the chemiluminescent signals in the membrane were obtained using a ChemiDoc™ XRS System (BioRad). Densitometry was performed using Image J software.

### Immunofluorescence microscopy

Indirect immunofluorescence microscopy was performed as described previously [23]. Briefly, cells were seeded onto 12-mm glass coverslips coated with fibrinogen and collagen and cultured at 37°C for 24 h after siRNA transfection. After 24 h of low-temperature (27°C) culturing, cells were incubated at 27°C for 1 h before fixation with 4% paraformaldehyde in PBS and permeabilized with 0.1% Triton X-100 in PBS for 10 min, washed 3 times for 2 min each with PBS, and then blocked with 2.5% goat serum in PBS. Cells were incubated with primary antibodies diluted in blocking solution for 2 h. Following 3 washing steps with PBS and 0.2% Tween 20, the Alexa Fluor-labeled secondary antibodies (1:200) were applied and incubated 45 min and the coverslips mounted on slides with Vectashield/DAPI (Vector Labs). Microscopy was performed using Leitz epifluorescence microscope. Images were obtained with a cooled, charge-coupled high-resolution camera (Photometrics). IpLab Spectrum software (Signal Analytics) was used for image acquisition. For the ammonium chloride experiments, cells were temperature-rescued for 24 h before they were transferred to 37°C and incubated with growth medium containing 5 mM NH_4_Cl for 1 h followed by indirect immunofluorescence microscopy using CFTR and mannose-6-phosphate receptor (M6PR) antibodies.

### Biotinylation and endocytosis of cell surface CFTR

The expression of cell surface CFTR was measured after biotin-labeling using EZ-link^®^ Biotin-LC-hydrazide as described previously [28]. Following siRNA transfection and low temperature-rescue, cell surface CFTR was labeled by biotinylation, immunoprecipitation using anti-CFTR antibody (24–1) and then blotted with Avidin D-HRP. rΔF508 CFTR endocytosis was measured in a two-step labeling experiment as described previously [28]. rΔF508 CFTR endocytosis was calculated by the reduction of biotinylated CFTR after a 37°C warm-up period compared to the no warm-up control sample (0 time point) [28].

### Measurement of cell-surface half-life of rescued ΔF508-CFTR

The cell-surface half-life of rΔF508 CFTR was measured as previously described [29]. Following siRNA transfection and culturing the cells at 37°C for 48 h, CFBE41o-ΔF cells were incubated for an additional 48 h at 27°C to promote ΔF508 CFTR cell surface rescue. During the last 16 h at 27°C, the cells were preincubated with 150 μg/ml of cycloheximide. The cells were then cultured in fresh media containing cycloheximide and incubated at 37°C for 0, 1, 2, 4 and 6 h as indicated. At the end of incubation periods, the cell-surface CFTR was biotinylated using EZ-link^®^ Biotin-LC-hydrazide as described previously [28]. CFTR was immunoprecipitated from cell extracts using anti-CFTR antibody (24–1) and detected by blotting with avidin D-conjugated horseradish peroxidase and chemiluminescence.

### Ussing chamber analyses

Short-circuit currents (*I*_*SC*_) were measured under voltage clamp conditions using MC8 voltage clamps and P2300 Ussing chambers (Physiologic Instruments, San Diego, CA) as previously described [23]. CFBE41o-ΔF monolayers were initially bathed on both sides with identical Ringer’s solutions containing (in mM) 115 NaCl, 25 NaHCO_3_, 2.4 KH_2_PO_4_, 1.24 K_2_HPO_4_, 1.2 CaCl_2_, 1.2 MgCl_2_, and 10 D-glucose (pH 7.4). Solutions on both sides were vigorously stirred by bubbling through 95%O_2_:5% CO_2_ gas. Short-circuit current measurements were obtained using an epithelial voltage clamp. A one-second 3-mV pulse was imposed every 10 s to calculate the resistance by Ohm’s law. Where indicated, the mucosal solution was changed to a low Cl^-^ solution containing 1.2 mM NaCl and 115 mM Na^+^ gluconate, and all other components as above. Amiloride (100 μM) was added to block residual Na^+^ current, followed by the CFTR agonist forskolin (20 μM) and potentiator genistein (50 μM) as indicated. CFTR_Inh_-172 (10 μM) was added to the apical solution at the end of experiments to block CFTR-dependent *I*_*SC*_. All chambers were maintained at 37°C during experiments.

### Measurement of the ubiquitin levels on rescued ΔF508 CFTR

CFBE41o-ΔF cells were transfected with specific siRNA oligos to deplete adaptor protein or E3 ubiquitin ligases and cultured at 37°C for 48 h. The cells were then switched to 27°C for 48 h to rescue ΔF508 CFTR to the cell surface. During the last 16 h, the cells were treated with 150 μg/ml cycloheximide. The immature form of intracellular ΔF508 CFTR (b-band) was degraded as described previously ([11], data not shown). The cells were then incubated with fresh media containing cycloheximide at 37°C for the time periods indicated to promote endocytosis. To block the endocytosis, 5 μM of Dyngo 4A was added to inhibit the dynamin-mediated internalization. CFTR from the cell lysates were immunoprecipitated using anti-CFTR antibody (24–1) and the ubiquitin level on the CFTR molecules was measured by blotting with a polyclonal anti-ubiquitin antibody (Millipore, Cat. # 09–408, 1:1000 dilution).

### Data analysis and statistics

All experiments were repeated at least 3 times in duplicate, and the data were expressed as the mean ± standard error. Statistical analysis was performed using student’s *t* test (2-tailed) in Microsoft Excel (Seattle, WA) and significance was determined at the p<0.05 levels.

## Results

### siRNA depletion of AP-2 (μ2 subunit) and Dab2 increases the surface pool of low-temperature rescued ΔF508 CFTR

**Fig 1:**
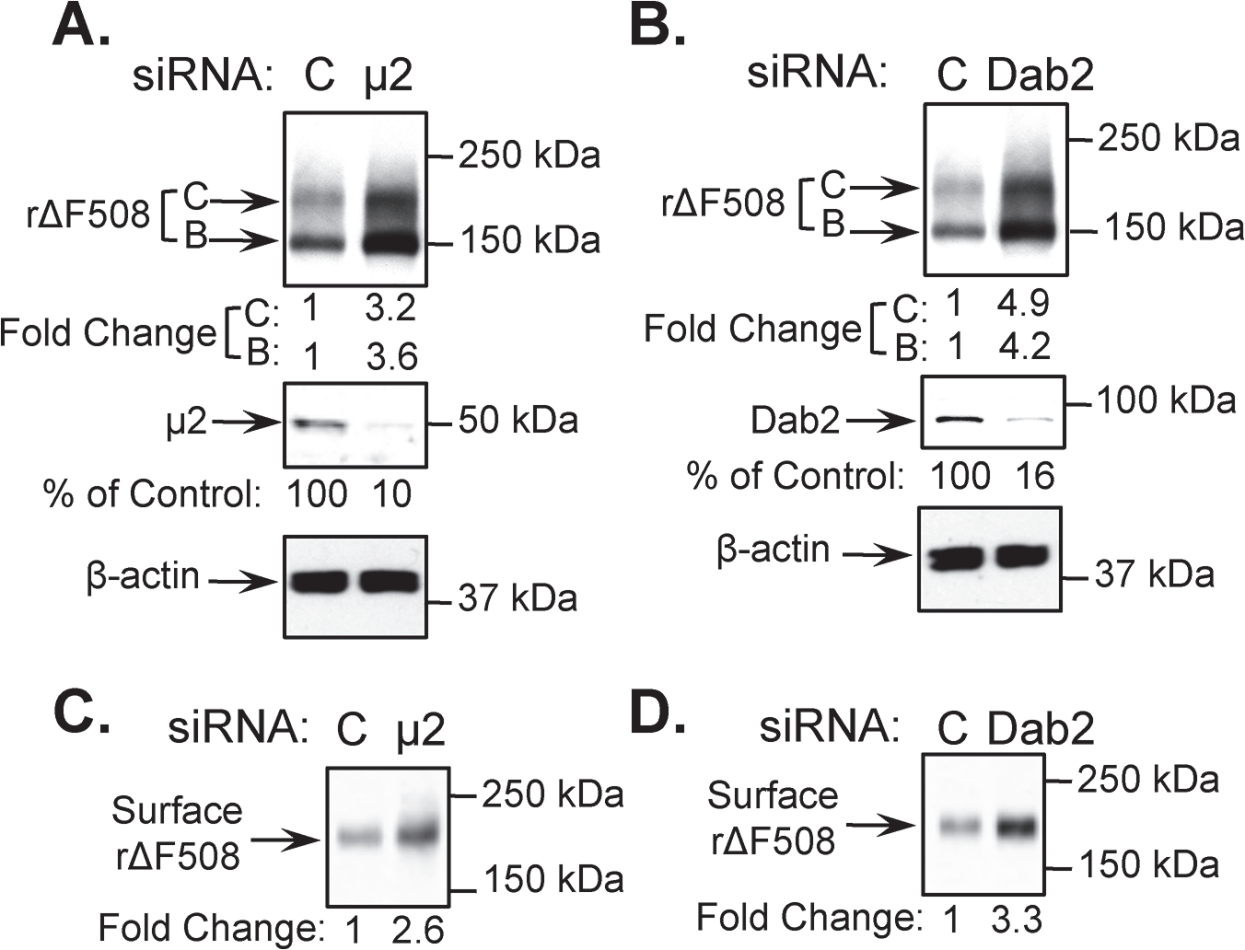
Increased cell surface expression of rescued ΔF508 CFTR (rΔF508 CFTR) following μ2 and Dab2 depletion. The consequences of μ2 depletion and Dab2 depletion on total ΔF508 CFTR expression (**A** and **B**) and on surface rΔF508 CFTR expression (**C** and **D**). CFBE41o-ΔF cells were transfected with 20 nM siRNA duplexes targeted specifically to μ2 or Dab2 or control (C) siRNA. 48 h after transfection, the control and siRNA depleted cells were cultured for an additional 24 h at 27°C to promote ΔF508 CFTR delivery to the cell surface. 72 h after transfection, 25 μg of cell lysates were separated by SDS-PAGE and immunoblotted using anti-CFTR, anti-μ2 and anti-Dab2 antibodies. β-actin was blotted as loading control. The changes in μ2, Dab2 and rΔF508 CFTR levels following siRNA depletion are indicated below the blots. Cell surface expression of rΔF508 CFTR was monitored by biotinylation as described in the Experimental section. The molecular mass in kDa is indicated on the right-hand side of each panel.

To monitor loss of the surface pool of rΔF508 CFTR, we first examined the role of two adaptor complexes on the steady-state levels of rΔF508 CFTR after low temperature rescue. To accomplish this, we performed siRNA knockdown (KD) experiments on the μ2 subunit of AP-2 and Dab2 in human airway epithelial cells expressing ΔF508 CFTR (CFBE41o-ΔF). μ2, one of the four subunits of the AP-2 complex, is known to be required for AP-2 complex formation and function [30]. For the depletion experiments, we determined the maximum depletion conditions for each adaptor. After siRNA knockdown (KD) for 48 h, we cultured the cells for an additional 24 h at 27°C to facilitate ΔF508 CFTR surface expression. The results in Fig 1 illustrate that the μ2 KD efficiency was 90% (Fig 1A) and the Dab2 KD efficiency was 84% (Fig 1B). The amount of maturely glycosylated (C band) rΔF508 CFTR increased ~3 fold in the μ2 KD cells versus ~5 fold in the Dab2 KD cells compared to the control siRNA KD cells (C) (Fig 1A and 1B, respectively). The increase of rΔF508 CFTR after Dab2 KD is consistent with previous studies [26]. Significantly, the level of the core glycosylated ΔF508 CFTR (B band) was also increased proportionately after μ2 or Dab2 KD in comparison to the siRNA control, indicating that the depletion of the adaptors might affect intracellular protein trafficking as shown previously [31, 32]. In addition, using cell surface biotinylation, we compared the surface pools of rΔF508 CFTR following KDs of μ2 and Dab2. We found a ~3-fold increase in cell surface rΔF508 CFTR following the KD of each adaptor compared to control conditions (Fig 1C).

### AP-2 (μ2) and Dab2 are necessary for efficient rΔF508 CFTR endocytosis

**Fig 2:**
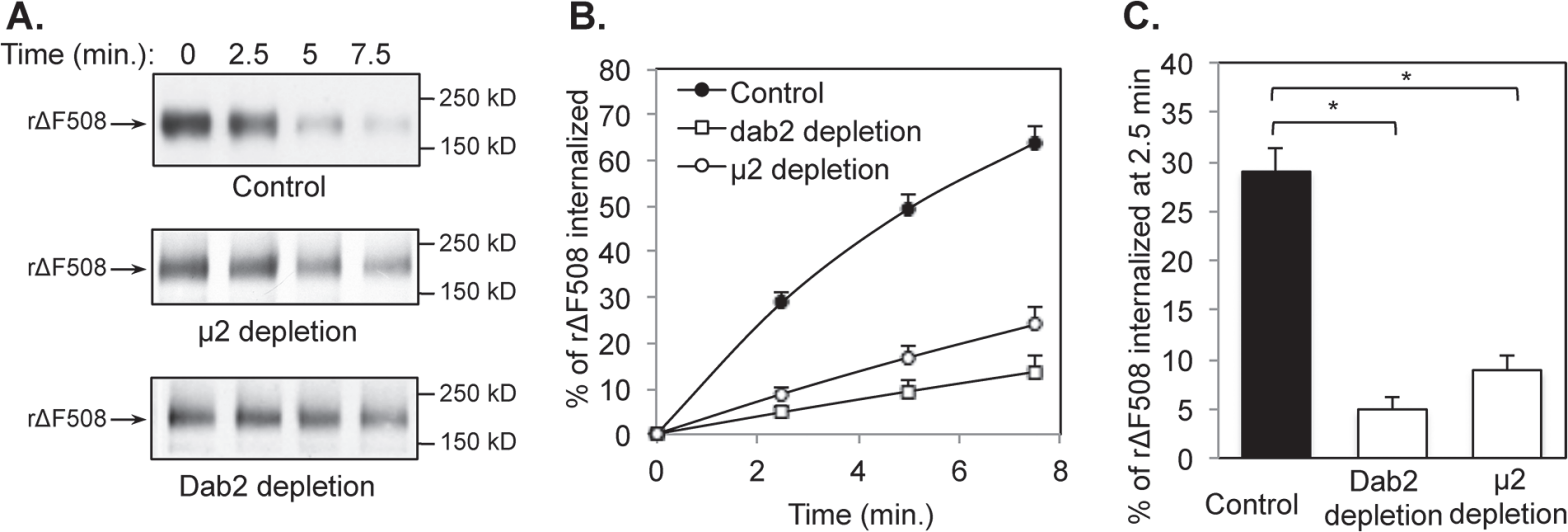
Reduced endocytosis rates of rΔF508 CFTR following μ2 or Dab2 depletion. CFBE41o-ΔF cells were transfected with control, μ2 or Dab2 siRNA oligonucleotides as indicated. At 24 h after transfection, the cells were transferred to Transwell filters and incubated for an additional 4–5 days under an air-liquid interface. During the last 24 h, the cells were incubated at 27°C to promote ΔF508 CFTR rescue. The efficiency of μ2 and Dab2 depletion was >90%. CFTR internalization assays were performed as described previously [28]. **(A)** Representative gels of CFTR internalization assays. The molecular mass in kDa is indicated on the right-hand side. **(B)** Quantitative analysis of rΔF508 CFTR internalization rates during a 7.5 min time period. The percentage of internalized CFTR was calculated from the loss of biotinylated CFTR during a 37°C incubation for time periods indicated after comparing to that at time 0 min under each condition (n = 3). **(C)** Quantitative analysis of CFTR internalization rates after 37°C warm-up for 2.5 min following μ2 or Dab2 depletion. Depletion of μ2 or Dab2 significantly reduced CFTR internalization rates in a 2.5 min time period (n = 3, *p<0.05).

Since the surface rΔF508 CFTR expression levels were affected by the KDs, we next tested if both adaptors were necessary for rΔF508 CFTR endocytosis. For these experiments, we depleted the cells of μ2 or Dab2 and cultured cells as polarized monolayers on filter supports for an additional 4 to 5 days under an air/liquid interface. We monitored rΔF508 CFTR endocytosis at 37°C after it was rescued to the cell surface in low temperature using a surface biotinylation assay (see Materials and Methods section). The level of μ2 or Dab2 depletion was ~90% as measured by immunoblotting (data not shown). The results shown in Fig 2 indicate that both μ2 and Dab2 are required for rΔF508 CFTR endocytosis. The rate of rΔF508 CFTR internalization was reduced in either μ2 or Dab2 depleted cells in comparison to the control during the 7.5 min warm-up at 37°C (Fig 2B). Within 2.5 min warm-up, ~29% of cell surface rΔF508 CFTR was internalized in the control cells. In contrast, only ~4.5% and 8.9% of cell surface rΔF508 CFTR molecules were internalized during the 2.5 min warm-up periods in cells depleted with Dab2 and μ2 respectively (Fig 2C). These results indicate that both μ2 and Dab2 are required for rΔF508 CFTR endocytosis since the KDs result in ~69% and ~83% decreases in endocytosis respectively when compared to the control.

### Dab2 depletion increases the surface half-life and chloride channel activity of rΔF508 CFTR

**Fig 3:**
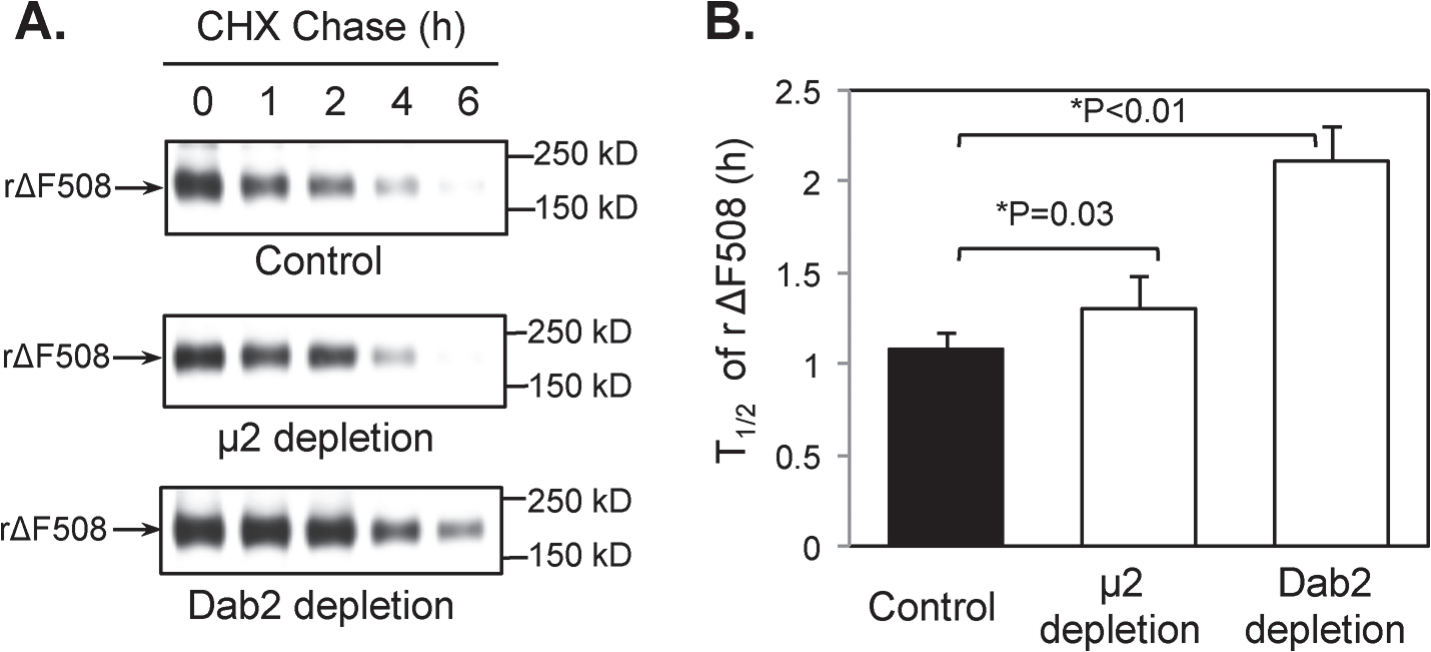
Increased cell surface half-life of rΔF508 CFTR in μ2- and Dab2-depleted cells. CFBE41o-ΔF cells were transfected with control, μ2, or Dab2 siRNA oligonucleotides. 48 h after transfection, the cells were cultured for 24 h at 27°C to allow cell-surface expression of rΔF508 CFTR. Cell surface rΔF508 CFTR was then monitored by biotinylation as described in the Material and Methods section after incubating with cycloheximide (CHX)-containing medium at 37°C for time periods indicated. Representative gels are shown (**A**) and quantitative analysis of the half-lives of rΔF508 CFTR under each experimental condition is shown (**B**). Dab2 depletion resulted in a ~2 fold increase in the half-life of cell surface rΔF508 CFTR (n = 3).

Since rΔF508 CFTR endocytosis was dramatically affected by the KD of μ2 or Dab2, we next tested if either of the adaptors were important for the rapid degradation of rΔF508 CFTR at 37°C by monitoring the cell surface half-life of rΔF508 CFTR. After siRNA KD of each of the complexes and low temperature correction, we switched the cells to 37°C during a cycloheximide blockade and monitored loss of the biotinylatable surface CFTR over time. The results shown in Fig 3 indicate that the surface half-life of rΔF508 CFTR with the control siRNA is 1.1 ± 0.1 h, which is consistent with previous reports (29). The siRNA KD of μ2 had a modest effect on rΔF508 CFTR half-life (1.3 ± 0.2 h), while KD of Dab2 had a more pronounced effect (2.1 ± 0.2 h). The results indicate that μ2 and Dab2 are both necessary for rΔF508 CFTR endocytosis, whereas Dab2 appears to be also important in the post-endocytic down-regulation of rΔF508 CFTR.

**Fig 4:**
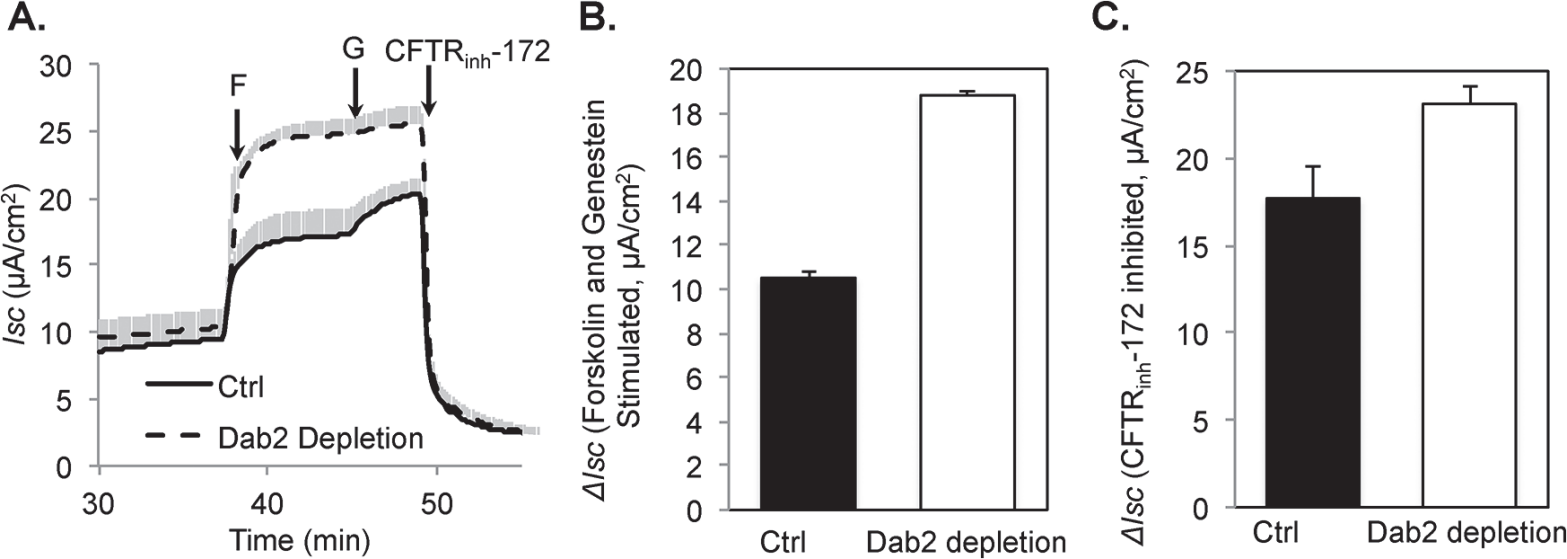
Increased transepithelial chloride transport following Dab2 depletion in CFBE41o-ΔF monolayers. CFBE41o-ΔF cells were transfected with control or Dab2 siRNA oligonucleotides. At 24 h after transfection, cells were lifted, seeded on to Transwell filters and cultured for an additional 4–5 days. The last 24 h, the cells were cultured at 27°C to promote ΔF508 CFTR delivery to the cell surface. The *I*_*SC*_ across the monolayers was measured in Ussing chambers as described in the Experimental Section. **(A)** Representative tracings from control and Dab2-depleted monolayers. After a stable baseline was attained, 20 μM forskolin (F), 50 μM genistein (G) and 10 μM CFTR_inh_-172 were added at the indicated arrows. **(B)** Forskolin and genistein activated *I*_*SC*_. *ΔI*_*SC*_ was calculated as an increase in *I*_*SC*_ after forskolin and genistein addition over the base-line currents (n = 4). **C.** CFTR_inh_-172 inhibited *I*_*SC*_. *ΔI*_*SC*_ was calculated as a decrease in *I*_*SC*_ after CFTR_inh_-172 addition.

At the cell surface wild type CFTR is in a functional complex [33], whereas rΔF508 CFTR is unstable and rapidly degraded [8–10, 12]. Given that Dab2 KD enhanced the surface stability of rΔF508 CFTR, we next tested how the chloride channel activity is affected during the Dab2 KD. In these experiments, we first depleted Dab2 or treated with a control siRNA and 24 h later plated the cells onto filter supports and grew them at an air/liquid interface for 5 days. During the last 24 h the cells were cultured at 27°C to promote ΔF508 CFTR rescue. The monolayers were then mounted in Ussing chambers and analyzed. Forskolin was added to elevate intracellular cAMP levels and activate rΔF508 CFTR. Genistein was added to maximally stimulate the channels and to monitor the non-cAMP activated component of rΔF508 CFTR activity (Fig 4A). The forskolin and genistein stimulated currents were roughly double in the Dab2 depleted monolayers compared to the controls (Fig 4B; 18.8 ± 0.2 compared to 10.5 ± 0.3 μA/cm^2^, respectively). The change in *I*_*sc*_ with the CFTR specific inhibitor, CFTR_inh_-172, was also greater in Dab2 depleted monolayers (23.1 ± 1.0 compared to 17.7 ± 1.9 μA/cm^2^, respectively; Fig 4C), although the difference was less pronounced because the Dab2 depleted cells had higher baseline currents, suggesting some degree of pre-activation prior to forskolin and genistein treatment. These results are consistent with elevated surface expression of rΔF508 CFTR during Dab2 depletion, but do not imply that Dab2 depletion increases channel activity through any other mechanism besides simply elevating the surface expression of rΔF508 CFTR.

### Depletion of c-Cbl, CHIP, and Nedd4-2 do not affect rΔF508 CFTR endocytosis

**Fig 5:**
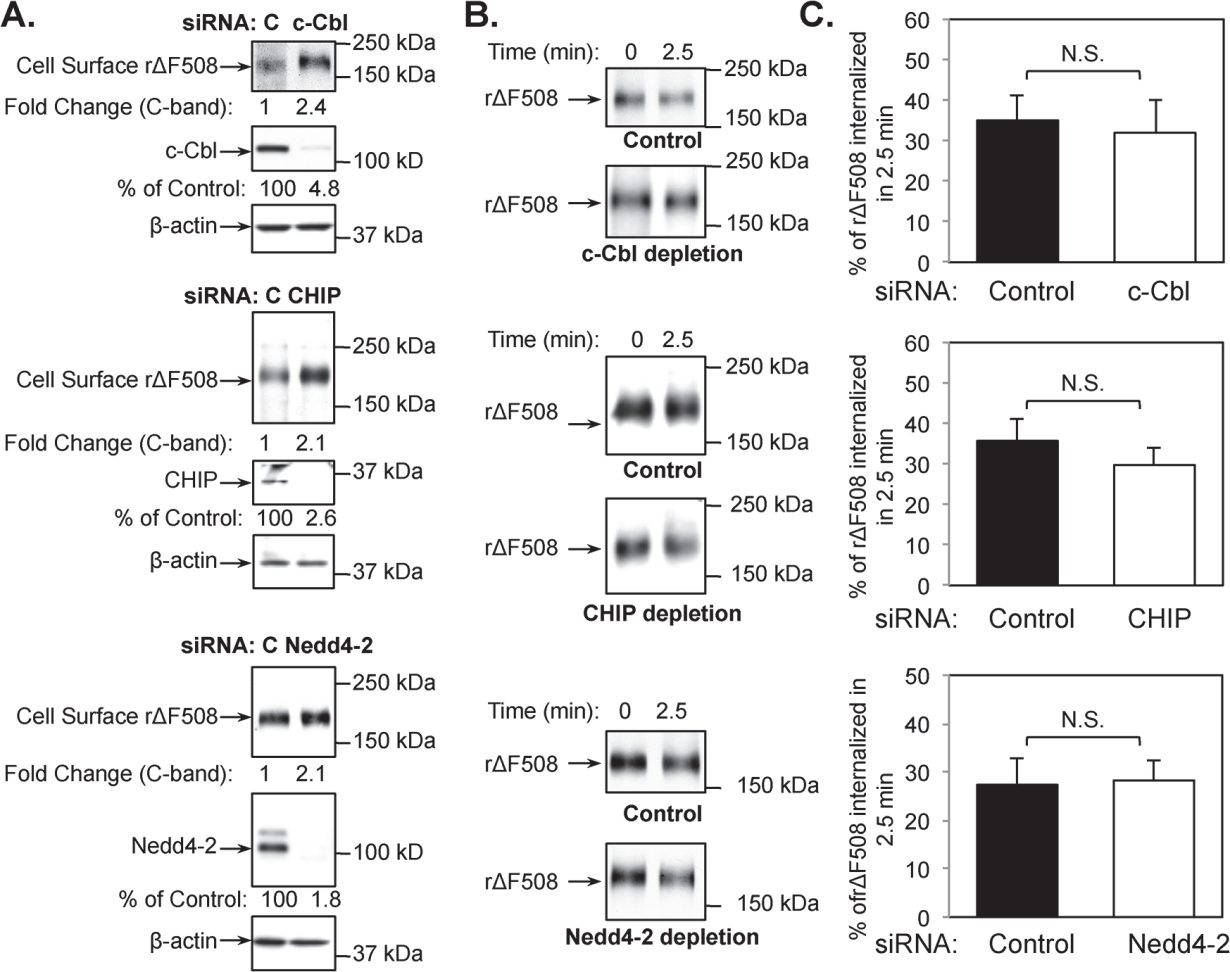
c-Cbl, CHIP or Nedd4-2 depletion do not affect rΔF508 CFTR endocytosis. CFBE41o-ΔF cells were transfected with control or c-Cbl, CHIP, or Nedd4-2 siRNA oligonucleotides. At 24 h after transfection, cells were transferred to Transwell filters and cultured for an additional 4–5 days. The last 24 h, the cells were cultured at 27°C to promote ΔF508 CFTR delivery to the cell surface. **(A)** Cell surface expression of rΔF508 CFTR after siRNA transfection of control (C), c-Cbl, CHIP or Nedd4-2 as indicated. The knockdown efficiency of c-Cbl, CHIP, and Nedd4-2 was >95%. β-actin was blotted as a loading control. **(B)** Representative blots showing the remaining surface rΔF508 CFTR after 2.5 min internalization at 37°C. **(C)** Quantitative analysis of rΔF508 CFTR internalization after 2.5 min warm-up following c-Cbl, CHIP, or Nedd4-2 siRNA depletion and low-temperature rescue. The rate of rΔF508 CFTR internalization was measured after 2.5 min warm-up as described in the Material and Methods section. Depletion of c-Cbl, CHIP, or Nedd4-2 had no significant (N.S.) effect on rΔF508 CFTR internalization (n = 3).

A number of E3 ligases have been implicated in the down-regulation of wild type or rΔF508 CFTR from the cell surface [11, 17, 34, 35], but whether they affect endocytosis or degradation or both is unclear. Here we separated these two processes and monitored the role of c-Cbl, CHIP, and Nedd4-2 in rΔF508 CFTR endocytosis. Each of the E3 ligases was depleted using siRNA, resulting in more than 90% reduction in their protein levels (Fig 5A). Cell surface levels of rΔF508 CFTR were then tested for each of the KDs and internalization assays were performed on polarized monolayers as described above. The results shown in Fig 5 indicate that the surface pools slightly increased (~2 fold) following the depletion of each of the E3 ligases. The E3 KDs increased the cell surface pools by increasing the amount of protein that may reach the cell surface. All three knockdowns increased CFTR B band and thus allowed for more protein to be rescued by low temperature (data not shown), which is consistent with the results shown by Caohuy et al. in CFPAC-1 cells [34]. More importantly, depletion of each of the E3 ligases had little (CHIP) or no effect (c-Cbl and Nedd4-2) on rΔF508 CFTR endocytosis (Fig 5B and 5C). CHIP depletion did show a modest effect on the endocytosis rate (~17% decrease compared to the control, P = 0.07), but it certainly wasn’t comparable to Dab2 or μ2 depletion. The results suggest that ubiquitination at the cell surface by these E3 ligases do not play a significant role in rΔF508 CFTR endocytosis in polarized human airway epithelial cells.

### CHIP regulates the surface half-life of rΔF508 CFTR

**Fig 6:**
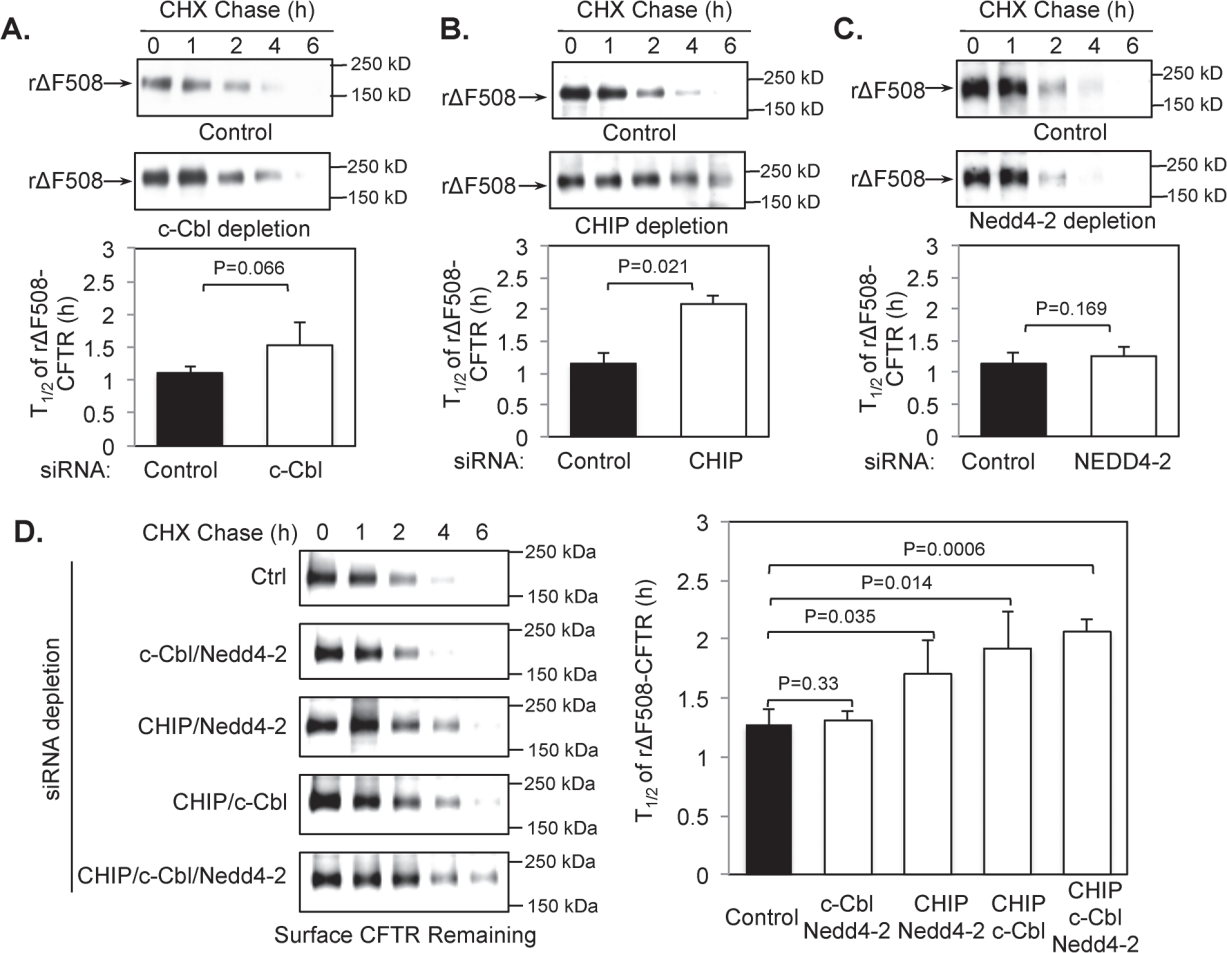
CHIP depletion increases the surface half-life. CFBE41o-ΔF cells were transfected with control or c-Cbl **(A)**, CHIP **(B),** or Nedd4-2 **(C)** siRNA oligonucleotides and cultured for 48 h at 37°C. The cells were then transferred to 27°C and incubated for an additional 48 h. Protein synthesis was stopped by preincubation with cycloheximide during the last 2 h at 27°C. Fresh media containing cycloheximide (CHX) was added and the cells were incubated for 0 to 6 h at 37°C and the surface pool of rΔF508 CFTR was detected using biotinylation as described in the Material and Methods section. Quantitative analysis of the blots is shown at the bottom (n = 3). The CHIP depletion significantly increased the cell surface half-life of rΔF508 CFTR, whereas c-Cbl depletion had little and Nedd4-2 depletion had no effect. **(D)** Cell surface half-life of rΔF508 CFTR was analyzed after CHIP depletion in combination with c-Cbl and/or Nedd4-2 as indicated. Depletion of c-Cbl and Nedd4-2 in combination with CHIP depletion did not have additional effect on the surface half-life of rΔF508 CFTR.

Next, we tested the effect of depletion of each of the E3 ligases on the surface stability of rΔF508 CFTR. To do this, we first transfected the CFBE41o- ΔF cells with siRNA oligos targeting each E3 ligase followed by low-temperature rescue of ΔF508 CFTR. The cell surface half-life of rΔF508 CFTR was determined at 37°C by biotinylation experiments as descried in the Experimental Methods section after cycloheximide block of protein synthesis. Fig 6 shows that ΔF508 CFTR rescued to the cell surface in the siRNA control sample was rapidly degraded after switching to 37°C, with a half-life ~1.1 h. siRNA depletion of c-Cbl increases the surface half-life to 1.5 ± 0.3 h (Fig 6A), although this change was not significant (P = 0.066). siRNA KD of CHIP had a more pronounced effect and changed the half-life to 2.1 ± 0.6 h (P = 0.02), suggesting that CHIP was required for rΔF508 CFTR down-regulation (Fig 6B). And finally, KD of Nedd4-2 had no significant effect on rΔF508 CFTR since the half-life was not affected (1.1 ± 0.2 versus 1.3 ± 0.1 h; Fig 6C (P = 0.169)).

**Fig 7:**
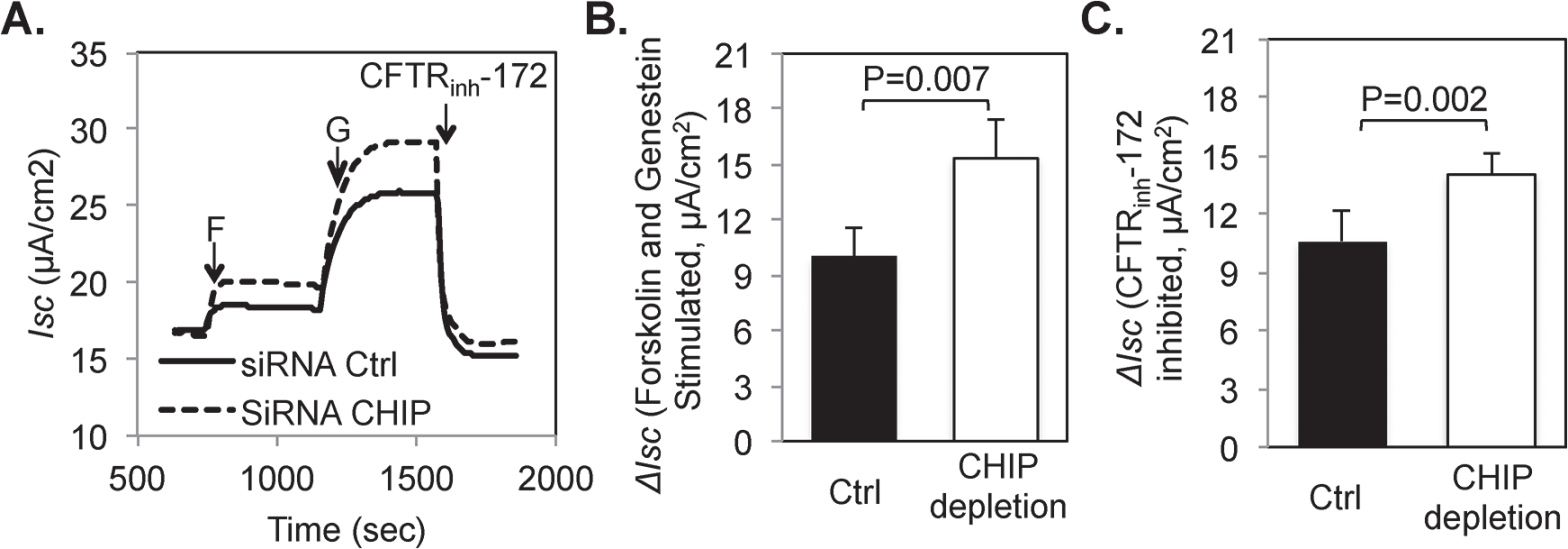
CHIP Depletion increases the function of rΔF508 CFTR. CFBE41o-ΔF cells were transfected with control or CHIP siRNA oligonucleotides, seeded on to Transwell filters and the *I*_*SC*_ across the monolayers was measured in Ussing chambers as described in Fig 4 legend. **(A)** Representative tracings from control and CHIP-depleted monolayers. After a stable baseline was attained, 20 μM forskolin (F), 50 μM genistein (G) and 10 μM CFTR_inh_-172 were added at the indicated arrows. **(B)** Forskolin and genistein activated *I*_*SC*_. *ΔI*_*SC*_ was calculated as an increase in *I*_*SC*_ after forskolin and genistein addition over the base-line currents. **C.** CFTR_inh_-172 inhibited *I*_*SC*_. *ΔI*_*SC*_ was calculated as a decrease in *I*_*SC*_ after CFTR_inh_-172 addition. (n = 6)

To determine if combinations of the E3 ligases had additive effects, we measured the cell-surface half-life of rΔF508 CFTR after KD of two or all three E3 ligases. The results shown in Fig 6D demonstrate that the depletion of c-Cbl and/or Nedd4-2 in addition to CHIP KD has no additional effect on the degradation of rΔF508 CFTR. Based on these results, we conclude that the CHIP E3 ligase plays a major role in the surface instability of rΔF508 CFTR in airway epithelial cells. In agreement with the increased half-life of rΔF508 CFTR, the CFTR channel function was also enhanced following CHIP depletion as determined by Ussing chamber experiments (Fig 7A). The forskolin and genistein stimulated currents (*ΔI*_*sc*_) were increased from 9.97 ± 1.54 μA/cm^2^ in the control to 15.33 ± 2.06 μA/cm^2^ in the CHIP depleted monolayers. Further, the CFTR_inh_-172 inhibited *ΔI*_*sc*_ also increased by ~40% in CHIP depleted monolayers (Fig 7B).

### Dab2 depletion inhibits rΔF508 CFTR delivery to the late endosome

**Fig 8:**
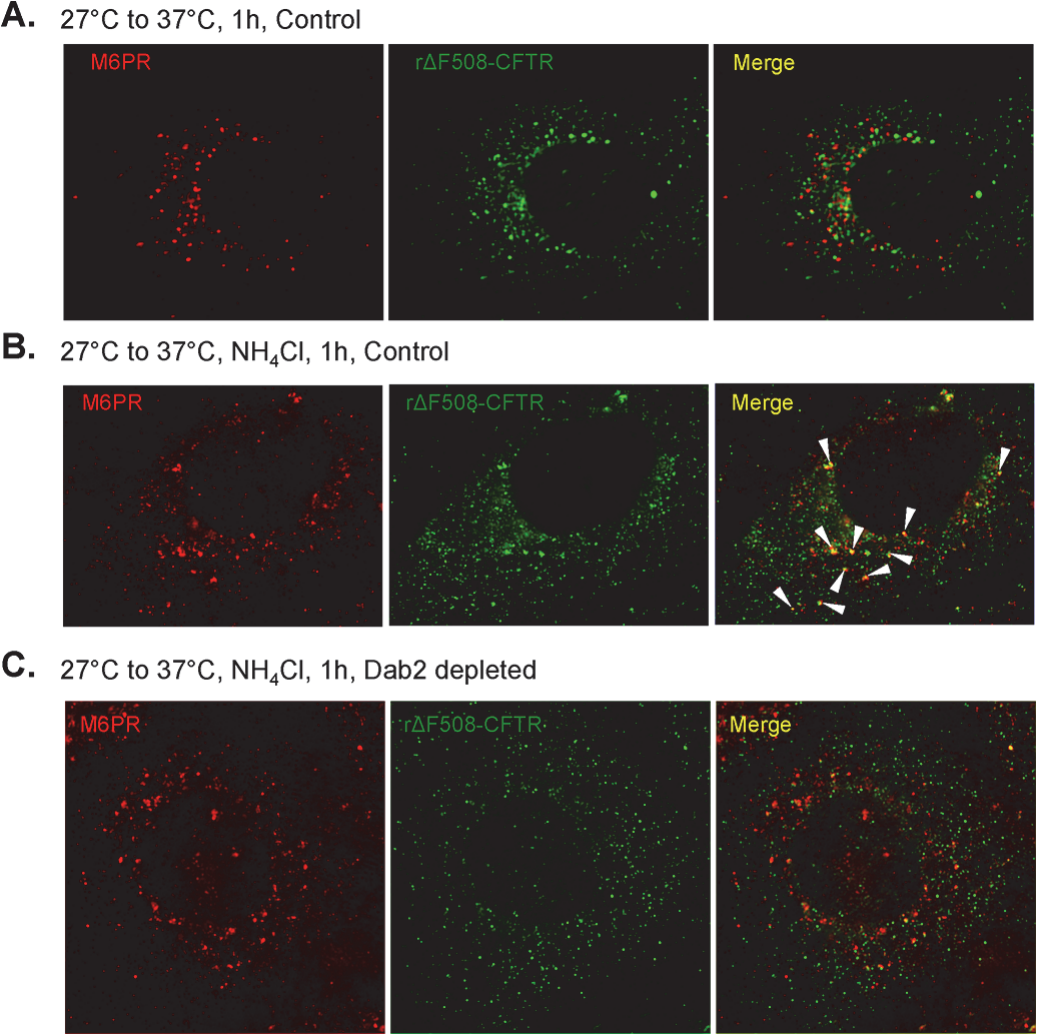
rΔF508 CFTR delivery to the late endosomes is inhibited in Dab2-depleted cells. CFBE41o-ΔF cells were treated with control **(A** and **B)** or Dab2-specific **(C)** siRNA oligonucleotides. At 24 h after transfection, the cells were cultured at 27°C for an additional 48 h to facilitate cell surface delivery of ΔF508 CFTR. After the low-temperature rescue, one set of the control cells was transferred to 37°C for 1 h (A), another set of the control (B) and the Dab2-depleted cells (C) were treated with 5 mM ammonium chloride for 1 h at 37°C followed by immunofluorescent staining of CFTR and M6PR. **(A) r**ΔF508 CFTR and mannose-6-phosphate receptor (M6PR) do not co-localize in control untreated cells. **(B)** Ammonium chloride treatment (inhibition of lysosomal degradation) resulted in rΔF508 CFTR and M6PR co-localization (right-hand panel, arrowheads)**. (C)** Dab2 depletion and ammonium chloride treatment together enhanced rΔF508 CFTR staining; however, no co-localization of rΔF508 CFTR and M6PR is apparent.

Our results above show that both AP2 and Dab2 are involved in the internalization of rΔF508 CFTR from the cell surface, and Dab2 appeared to be important for removal of rΔF508 CFTR from the cell surface. To test this idea, we determined if rΔF508 CFTR delivery to the late endosome/lysosome was inhibited by Dab2 depletion. We depleted Dab2 from cells and cultured the cells at low temperature (27°C) to build up rΔF508 CFTR at the cell surface. Then we compared the distribution of rΔF508 CFTR in control siRNA treated cells and Dab2 depleted cells with a marker of the late endosomal compartment, the mannose-6-phosphate receptor (M6PR). Since lysosomal proteases are delivered to this compartment, proteolytic degradation of proteins begins here [36, 37]. To inhibit the degradation of rΔF508 CFTR in this compartment, we treated the cells with a weak base, ammonium chloride and visualized rΔF508 CFTR in the late endosomal compartment. We have used this technique to monitor transferrin receptor chimeras that were delivered to the lysosomal compartment [37] and wild type CFTR transit through the late endosome [23]. The results shown in Fig 8 demonstrate that under control conditions, rΔF508 CFTR and M6PR do not co-localize (Panel A, merge). However, when cells are treated with the weak base to inhibit proteolysis, co-localization occurs (Panel B, merge, arrowheads), suggesting that rΔF508 CFTR degradation occurs in M6PR-positive compartment. In contrast, Dab2 depleted cells treated with weak base, co-localization is not seen, suggesting that the Dab2 depletion has compromised rΔF508 CFTR delivery to the late endosome, and consistent with the increase in rΔF508 CFTR half-life measurements.

### Dab2 depletion increases the intracellular ubiquitinated rΔF508 CFTR pool that is CHIP-mediated

**Fig 9:**
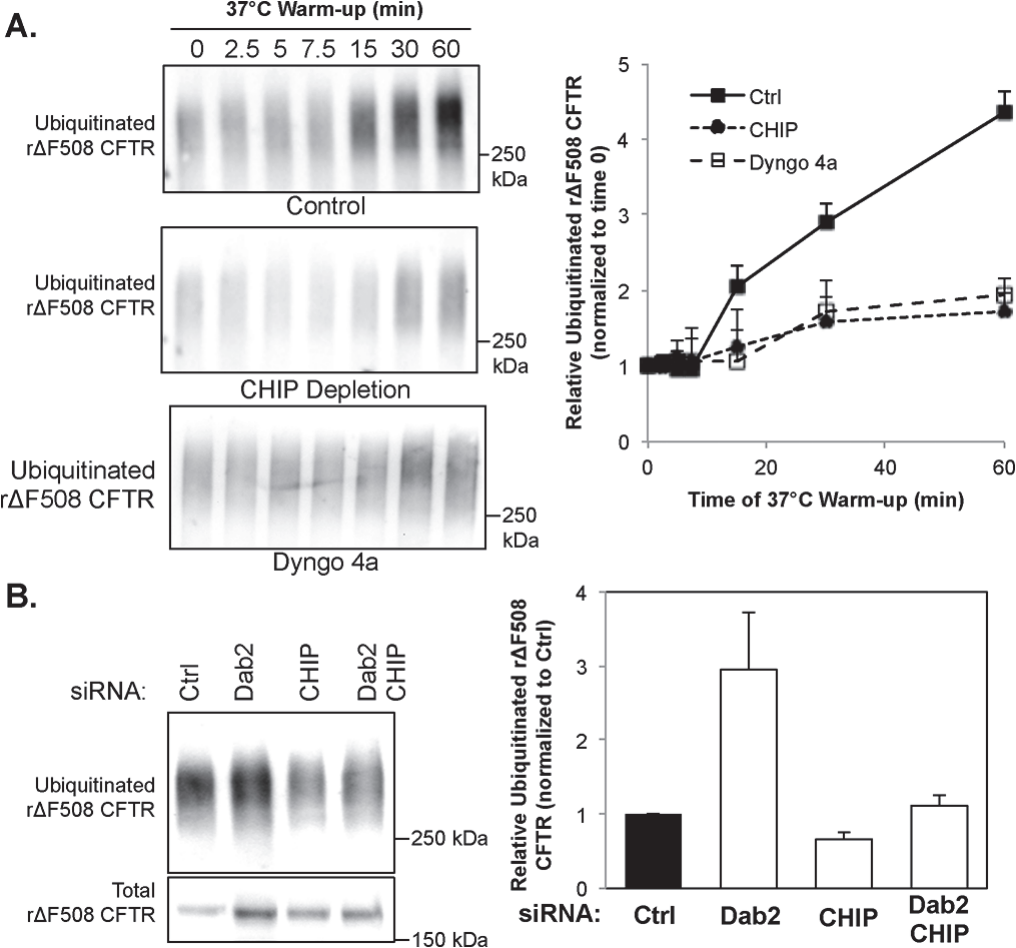
Ubiquitination of rΔF508 CFTR by CHIP occurs in the post-endocytic compartments. **(A)** CFBE41o-ΔF cells were transfected with control or CHIP siRNA oligonucleotides at 37°C and then incubated for 48 h at 27°C to rescue the ΔF508 CFTR to the cell surface. During the last 16 h of low-temperature incubation, 150 μg/ml of cycloheximide was added to the culture to inhibit protein synthesis and eliminate the internal biosynthetic pool of ΔF508 CFTR. The cells were then switched to 37°C to promote endocytosis of the rΔF508 CFTR for the time periods indicated. The CFTR was then pulled-down by immunoprecipitation with specific antibody (24–1) and blotted with an ubiquitin antibody. The ubiquitin level is increased after 15 to 60 min warm-up at 37°C incubator in control cells (upper panel). The increase of ubiquitinated rΔF508 CFTR was attenuated under CHIP depletion (middle panel). The lower panel shows that the ubiquitination of rescued ΔF508 CFTR was reduced in the presence of 5 μM Dyngo 4a, which inhibits endocytosis. The relative ubiquitin level of rΔF508 CFTR after 37°C warm-up was analyzed in control, CHIP depleted and Dyngo 4a-treated cells (n = 3). **(B)** The ubiquitinated pool of rΔF508 CFTR was analyzed after Dab2 or CHIP depletion as described above. Dab2 depletion increases the CHIP-mediated ubiquitination of rΔF508 CFTR by blocking the post-endocytic trafficking.

Our data above indicate that two sequential steps are involved in the instability of rΔF508 CFTR: the initial endocytosis of rΔF508 CFTR is mediated through the AP2 and Dab2 complexes and the later post-endocytic targeting of rΔF508 CFTR to the late endosomes and lysosomes is Dab2-dependent. To further delineate the role of Dab2 and the E3 ligases in the cell-surface turnover of rΔF508 CFTR, we measured how the ubiquitination of rΔF508 CFTR is affected. First, we tested how ubiquitination of rΔF508 CFTR takes place in control cells. CFBE41o-ΔF cells were cultured at 27°C for 48 h to rescue the ΔF508 CFTR to the cell surface. During the last 16 h of low-temperature culturing, 150 μg/ml cycloheximide was added to inhibit protein synthesis and deplete the internal pool of newly synthesized ΔF508 CFTR as described before [9]. The cells were then shifted to 37°C for different time periods to promote endocytosis. As shown in Fig 9A, the ubiquitin levels for rΔF508 CFTR remained constant during the first 7.5 min of 37°C warm-up and then began to increase at the 15 min time point in the control cells. We carefully monitored the temperature of the cells to insure that this lag period was not due to cell temperature effects. The ubiquitin level continued to rise after 15 min until the final time point (60 min). This increase of ubiquitination on rΔF508 CFTR was inhibited in the presence of Dyngo-4a, a dynamin inhibitor, suggesting that the majority of ubiquitin was added to rΔF508 CFTR after internalization. The ubiquitination of rΔF508 CFTR was also largely diminished after CHIP depletion (Fig 9A). Dab2 depletion increases the ubiquitinated pool of rΔF508 CFTR 3-fold. This effect was largely attenuated by CHIP depletion (Fig 9B). Our results suggest that the rΔF508 CFTR localized in the peripheral membrane is first internalized through AP2 and Dab2 and then ubiquitinated by CHIP and targeted to the late endosomes/lysosomes for degradation. Dab2 plays a role in both internalization and lysosomal targeting of rΔF508 CFTR during the peripheral quality control process.

### VX-809 increases the cell-surface stability of rΔF508 CFTR by inhibiting its post-endocytic ubiquitination

**Fig 10:**
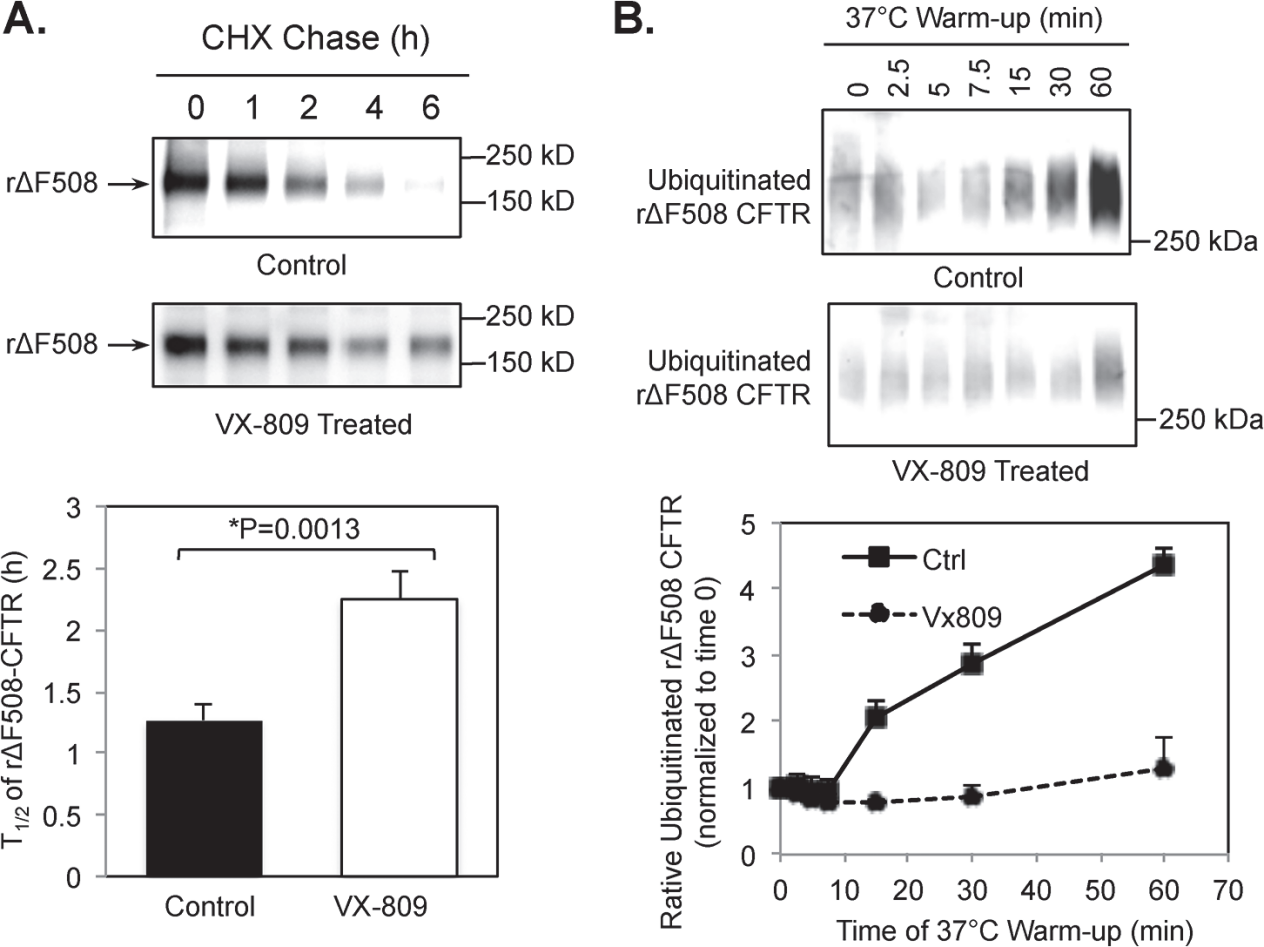
VX-809 stabilizes the cell-surface pool of rΔF508 CFTR. **(A)** ΔF508 CFTR in CFBE41o-ΔF cells were rescued to cell surface by incubating at 27°C for 48 h followed by 3 h pretreatment with 3 μM VX-809. The cells were transferred to fresh medium containing cycloheximide (CHX) with or without VX-809 that is prewarmed to 37°C and incubated at 37°C for the time periods indicated. The rescued ΔF508 CFTR (rΔF508) remained on the cell surface was measured by biotinylation as described in the Methods section. (B) The ubiquitination of rΔF508 CFTR were determined as described in Fig 9 legend. Treatment with 3 μM VX-809 inhibited the ubiquitination of rΔF508 CFTR.

Next, we tested whether the CFTR corrector VX-809 has any effect on stabilizing rΔF508 CFTR on the cell surface. To do this, CFBE41o^-^ cells expressing ΔF508 CFTR were first cultured for 48 h at 27°C to rescue ΔF508 CFTR to the cell surface. The cells were then transferred to 37°C culture with or without VX-809 and the rΔF508 CFTR molecules that remained on the cell surface were analyzed by biotinylation. As shown in Fig 10A, VX-809 increased the cell-surface half-life (t_1/2_) ~2 fold. Furthermore, the polyubiquitination of rΔF508 CFTR was reduced by VX-809 treatment (Fig 10B). These results demonstrated that the corrector VX-809 stabilizes the rΔF508 CFTR at the plasma membrane in addition to its function of promoting ΔF508 CFTR folding in the ER compartment.

## Discussion

CFTR molecules on the cytoplasmic membrane of epithelial cells are internalized to the endocytic compartments, from where they are either recycled back to the plasma membrane or sorted for lysosomal degradation. We and others previously identified the cell surface machinery responsible for wild type CFTR endocytosis and turnover [11, 18, 23]. The goal of the present studies was to identify critical components in the peripheral trafficking steps that contribute to the rapid turnover of the rΔF508 CFTR in human airway epithelial cells. The rationale is that these constituents can be potential targets to stabilize the mutant CFTR on the cell surface. Several lines of evidence suggest that peripheral quality control of rΔF508 CFTR occurs in post-endocytic compartments after internalization. First, the endocytosis of rΔF508 CFTR occurs rapidly with most of the channel internalized within 10 min, yet little if any of it appears to be ubiquitin modified. Second, polyubiquitinated rΔF508 CFTR begins to show up 15 min after internatization. Third, blocking endocytosis blocks rΔF508 CFTR polyubiquitination, lending further support that the ubiquitin is added at a subsequent step after endocytosis (Fig 9).

AP-2 and Dab2 were examined for their role on rΔF508 CFTR internalization and cell surface stability. We demonstrate that both proteins are important for rΔF508 CFTR endocytosis and this is similar to the results we found for wild type CFTR [23]. In addition, we found that siRNA knockdown of AP-2 adaptor complex had little effect on the protein half-life of wild-type and rΔF508 CFTR, whereas knockdown of Dab2 dramatically increased the half-lives of both proteins. Our results also indicate that the wild type and misfolded rΔF508 CFTR are sorted for endocytosis by the same machinery, but rΔF508 CFTR is degraded quickly, while the wild type protein remains stable. These results further suggest that the selection and rate-limiting step for CFTR removal from the cell periphery is not at the initial internalization, but rather at a later post-endocytic sorting step.

siRNA knockdown of either the AP-2 or Dab2 adaptor inhibits rΔF508 CFTR endocytosis. One explanation is that either adaptor interacts separately with rΔF508 CFTR. However, we do not believe that to be the case for the following reasons. First, the tyrosine-based signal (YDSI) found in the CFTR cytoplasmic tail, YDSI, has been shown to be critical for CFTR endocytosis [38], and μ2 has been shown to interact with this sequence [19–21]. Second, Dab2 uses a phosphotyrosine-binding domain (PTB) to bind to FXNPXY sequences found in the LDL receptor family members, and binds AP-2, phosphoinositides, and clathrin [39]. Dab2 has a marked preference for nonphosphorylated NPXY sequences [39], but no such sequence is present in CFTR. Third, if either adaptor mediates CFTR endocytosis independently, depletion of both should have an additive effect, but this is not the case for wild type CFTR [23]. Fourth, in intestinal epithelial cells, AP-2 interacts directly with CFTR, whereas Dab2 does not [22]. These data are consistent with the model in which CFTR interacts with a series of proteins beginning with AP-2, and AP-2 then interacts with Dab2, which recruits myosin VI. The present studies on rΔF508 CFTR endocytosis indicate that endocytosis of wild type and rΔF508 CFTR is mechanistically similar and that subsequent steps such as post-endocytic ubiquitination must be responsible for handling of misfolded rΔF508 CFTR. Analysis of a third adaptor molecule in the present studies, c-Cbl, indicated that it did not participate in rΔF508 or in wild type CFTR endocytosis [23], suggesting that c-Cbl is not a CFTR adaptor as suggested by Ye *et al*. [17].

Given the fact that cell surface proteins, particularly signaling receptors, are often ubiquitin modified prior to degradation and ΔF508 CFTR is rapidly removed from the cell surface at 37°C after low temperature rescue, we examined the role of ubiquitin ligases in the cell surface removal and degradation of rΔF508 CFTR. We chose three E3 ligases (c-Cbl, CHIP and Nedd4-2) implicated in CFTR degradation [11, 14, 17, 34]. Our data indicate that the CHIP ubiquitin ligase plays a major role in the down regulation of rΔF508 CFTR from cell surface. In contrast, the cell surface half-life of wild-type CFTR was not affected by CHIP depletion [23], suggesting that CHIP-mediated ubiquitination is a prerequisite for selectively degrading misfolded rΔF508 CFTR [23]. c-Cbl was previously shown to control wild type CFTR stability [17, 23], whereas CHIP depletion did not [23]. In HeLa and IB3 cells, CHIP has been shown to affect rΔF508 CFTR surface stability, whereas c-Cbl did not [11]. Interestingly, Nedd4-2 KD did not have any effect on rΔF508 CFTR surface stability. Koeppen *et al*. [35] demonstrated that wild type CFTR is not regulated by Nedd4-2 in human airway epithelial cells. Studies by Caohuy et al. [34] showed that Nedd4-2 KD in CFPAC-1 (pancreatic epithelial) and IB3-1 (bronchial epithelial) cells increased the biotinylatable surface pool and cell surface half-life of ΔF508 CFTR, suggesting that there are cell-type specific differences in the processing and/or trafficking of ΔF508 CFTR.

Analysis of these E3 ligases through KD studies shows no effect on rΔF508 CFTR endocytosis in spite of very efficient depletion. This data suggest that the 3 tested E3 ligases are not required for the endocytosis of rΔF508 CFTR. In our studies, we measured the dynamics of polyubiquitination of rescued ΔF508 CFTR. To do that, we first rescued ΔF508 CFTR to the plasma membrane by incubating at low temperature (27°C). The intracellular core-glycosylated ΔF508 CFTR was then eliminated by treating with cycloheximide as described [9]. The ubiquitin levels on the remaining rΔF508 CFTR were examined after incubating the cells at 37°C for 0 to 60 min. The results show that the ubiquitin modification of rΔF508 CFTR was dramatically increased after 15 to 60 min warm-up periods. The increased level of rΔF508 CFTR ubiquitination was reduced upon either CHIP KO or endocytosis inhibition. These results suggest that ubiquitination of rΔF508 CFTR occurs after endocytosis and is mediated by CHIP E3 ligase.

The post-endocytic trafficking of rΔF508 CFTR was affected by both Dab2 and CHIP KDs. Dab2 depletion interfered with delivery of ΔF508 CFTR to the late endosomal compartment and therefore appears to be important for both endocytosis and in the post-endocytic sorting. Given the role of myosin VI during the early steps in clathrin-mediated endocytosis and the transport step from sorting endosomes, as well as the known myosin VI-Dab2 interactions, [40–42], it is perhaps not surprising that Dab2 KD interferes with ΔF508 CFTR transport in the later transport steps. Therefore, Dab2 is not a part of the peripheral quality control machinery but is a critical component at several steps in the endocytic pathway including delivery to the late endosomes as shown by our studies. As a consequence, rescued ΔF508 CFTR was accumulated in the early endosomes, from where we propose that CFTR is ubiquitin modified by CHIP-mediated mechanisms.

VX-809, the most promising corrector, was shown to restore the membrane localization and function of ΔF508 CFTR to ~10% that of wild-type CFTR [43]. We tested whether VX-809 has any effects on the membrane stability of low-temperature (27°C) rescued ΔF508 CFTR that is presumed misfolded after switching to 37°C. Our results shown in Fig 10 demonstrated that VX-809 also enhances the half-life of misfolded ΔF508 CFTR on the membrane ~2 fold and decreases ubiquitination of rΔF508 CFTR. Our results are in consistent with other reports providing evidence that VX-809 extends the stability of ΔF508 CFTR on the plasma membrane [44, 45], possibly through direct binding of CFTR molecules as shown previously [46].

In summary, our data support the model in which both wild type and rΔF508 CFTR are internalized through clathrin-coated pits and internalization is mediated by AP-2 and Dab2. Therefore, we establish that enhanced internalization of rΔF508 CFTR is not responsible for the short cell surface function. Based on the results of our studies on both the wild type and mutant CFTR, we propose a model in which Dab2 serves as a bridging molecule rather than an adaptor by coupling AP-2 to the myosin VI-based machinery. Further, Dab2 is also necessary for the post-endocytic trafficking of rΔF508 CFTR, but is not a part of the peripheral quality control machinery dealing with rΔF508 CFTR. Analysis of 3 E3 ligases indicates that CHIP and c-Cbl, but not Nedd4-2 are important for ΔF508 CFTR lysosomal targeting in human airway epithelial cells. Our results suggest that the stability of misfolded ΔF508 CFTR at the peripheral cell surface is determined by CHIP-mediated ubiquitination in the post-endocytic compartments. A previous report demonstrated that the inactivation of CHIP E3 ligase increases the foldable ΔF508 CFTR in the ER [47]. These results suggest that inhibition of CHIP E3 ligase activity would increase the ER pool and stabilize the surface pool of ΔF508 CFTR. These additive effects could potentially provide an effective way to treat the majority of CF patients with ΔF508 mutation on CFTR. Furthermore, our results indicate inhibition of ΔF508 CFTR ubiquitination may have additional therapeutic effects when used in combination with a corrector molecule such as VX809. Just as importantly, this type of dual approach could be used with any CFTR mutant protein that is unstable at the cell surface.
